# Chaihu-Shugan-San Reinforces CYP3A4 Expression via Pregnane X Receptor in Depressive Treatment of Liver-Qi Stagnation Syndrome

**DOI:** 10.1155/2019/9781675

**Published:** 2019-10-31

**Authors:** Zehui He, Rong Fan, Chunhu Zhang, Tao Tang, Xu Liu, Jiekun Luo, Hanjin Cui, Yang Wang, Ruohuang Lu, Pingping Gan

**Affiliations:** ^1^Laboratory of Ethnopharmacology, Institute of Integrative Chinese Medicine, Xiangya Hospital, Central South University, 410008 Changsha, China; ^2^Department of Integrated Chinese and Western Medicine, Xiangya Hospital, Central South University, 410008 Changsha, China; ^3^Department of Stomatology, Third Xiangya Hospital, Central South University, 410008 Changsha, China; ^4^Department of Oncology, Xiangya Hospital, Central South University, 410008 Changsha, China

## Abstract

*Background*s. Chaihu-Shugan-San (CSS) is a classic traditional Chinese herbal prescription for treating depression. However, the underlying mechanism of the Chinese syndrome-specific efficacy of CSS is poorly understood. *Aim of the Study*. From traditional Chinese medicine and pharmacogenetics perspectives, the present study aimed to investigate the antidepressant effects of CSS on a mouse model of Liver-Qi Stagnation (LQS) syndrome and its underlying mechanisms. *Methods and Materials*. We used two main mouse models of depressive syndromes in the study, including LQS and liver stagnation and spleen deficiency (LSSD) syndrome. Tail suspension and forced swimming tests were used to evaluate the effects of CSS on animal behaviour. The expression level of the CYP450 enzyme from liver microsomes was analysed by western blot (WB) analysis and quantitative real-time polymerase chain reaction (qRT-PCR). More specifically, we analysed the key compounds of CSS that are responsible for CYP450 regulation via bioinformatics. Ultimately, luciferase assays were employed to confirm the prediction *in vitro*. *Results*. CSS remarkably reduced the immobile time in LQS rather than in LSSD mice. Although CSS significantly upregulated CYP2C9 in mice with both syndromes, activated translation of CYP3A4 induced by CSS was only observed in the LQS group. Bioinformatics analysis revealed that the unique regulation of CYP3A4 was responsible for the effects of glycyrrhetinic acid (GA) from CSS. Further luciferase assays confirmed the enhancement of CYP3A4 expression via the pregnane X receptor (PXR) pathway *in vitro*. *Conclusions*. CSS specifically upregulates the translation of CYP3A4 via the PXR pathway in depressed LQS mice. GA, a bioactive compound that originates from CSS, contributes to this activation. This work provides novel insight into Chinese syndrome-based therapy for depression.

## 1. Introduction

Depression is a prevalent psychiatric illness characterized by low mood, slow thought, and mental disorder. The World Health Organization (WHO) ranks depression as the leading contributor to global disability, affecting 322 million people [1–5]. Unfortunately, almost all antidepressants are monoaminergic agents, and 50–60% of patients treated with antidepressants have incomplete recovery with significant side effects or poor compliance [6, 7]. Therefore, neuroscientists and doctors are searching for adjunctive strategies to improve the clinical symptoms of depression [8, 9].

Chaihu-Shugan-San (CSS) is a classic prescription used in the clinic to treat depression [8]. CSS was initially described in a Chinese medical classic “yi-xue-tong-zhi” published in 1535 (Ming Dynasty of China). The formula mainly consists of seven crude herbs (the herbs and their component ratios are listed in Table 1). To date, CSS and its constituents have emerged as antidepressant agents for improving behaviour, increasing ERK5 activity, and modulating phospholipid and bile acid metabolism in mammalian models of depression [10–14]. Although abundant studies have reported the effects of CSS on depression, rodent models of chronic mild stress or unavoidable punishment (learned helplessness) have been adopted for inducing depressive symptoms [15]. According to the theory of traditional Chinese medicine (TCM), depression includes different syndromes, such as Liver-Qi Stagnation (LQS), a syndrome induced by abnormal flow of the liver qi, which is characterized by depression, chest tightness and sighing [16], and liver stagnation and spleen deficiency (LSSD), which includes poor digestion, weight loss, diarrhoea, a bad appetite, and so on [17]; these are the two main depressive syndromes, and each has its own specific symptoms. CSS is uniquely used to treat LQS. To our knowledge, no previous report has focused on the underlying mechanisms of the therapeutic specificity of TCM. Hence, further study is indispensable to elucidate the Chinese syndrome-specific efficacy of CSS in depression.

The cytochrome P450 (CYP450) family of enzymes, one of the cornerstones of pharmacogenetics, consists of key enzymes that metabolize drugs and other endo- or xenobiotics, including CYP1A2, CYP2C9, CYP2C19, CYP2D6, CYP2E1, and CYP3A4 [18–20]. Pharmacogenetics has become a key dimension of precision medicine to guide the application of certain antidepressants for individuals with interindividual variations of the expression and activity of CYP450 enzymes, which are regulated by the pregnane X receptor (PXR) [21]. As previously mentioned, we assume that every TCM syndrome of depression can be specifically described by changes in certain CYP450 enzymes, which partly account for the therapeutic differences of TCM syndromes of depression.

In this study, we aimed to observe whether CSS has specific efficacy for LQS of depression and to explore the underlying mechanisms from the perspective of CYP450 enzymes. Moreover, after performing bioinformatics analyses using the TCMSP, UniProt and DAVID databases, we searched for the potential bioactive compound that mainly targets CYP450 enzymes. Finally, we verified the prediction *in vitro*.

## 2. Material and Methods

### 2.1. Reagents

Dried crude herbs (Chaihu-Shugan-San, CSS) were purchased and identified in the pharmacy of Xiangya Hospital (Changsha, China). Voucher specimens (CH-NO-15021714, BS-NO-15021411, CP-NO-15020914, XF-NO-15021118, CX-NO-15022413, ZK-NO-15020706, GC-NO-15022715) were obtained and kept in the Laboratory of the Institute of Integrative Medicine, Central South University. Glycyrrhetinic acid (GA) was purchased from the Beijing Hengyuan Qitian Chemical Industry (Beijing, China). Rifampicin (RIF), pregnenolone 16*α*-carbonitrile (PCN), dimethyl sulfoxide (DMSO), and phenobarbital (PB) were obtained from Sigma-Aldrich (St. Louis, MO, USA). The reverse transcription system, dual-luciferase reporter assay system, pGL4.17-Luc, and pGEM-T constructs were supplied by Promega (Madison, WI, USA).

### 2.2. CSS Preparation

All herbs were processed into a lyophilized powder according to the established standard procedures [22]. Briefly, the herbs were boiled in distilled water at 100°C for 1 h twice. The combined supernatants of the two extracts were lyophilized (yield: 18.2%). Then, the powders were dissolved in distilled water to a final concentration of 0.03 g/mL before being used. Quality control of CSS was performed by UPLC (the details are provided in Supplementary [Supplementary-material supplementary-material-1]).

### 2.3. Animals

Seventy specific pathogen-free (SPF) Kunming male mice (20∼25 g) were obtained from the Experimental Animals Centre of Central South University, Changsha, Hunan. Mice were raised separately, 5 per cage, and were kept in rooms with a constant light–dark cycle (8:00 am–20:00 pm), room temperature (25°C), and 50 ± 10% relative humidity with free access to food and water. All animal experiments were performed following a protocol approved by the local Animal Ethics Committee of the Institution of Research Animal Care of Central South University with the principles of laboratory animal care (approval ID: 201303049).

### 2.4. Experimental Design and Drug Administration

**Figure 1:**
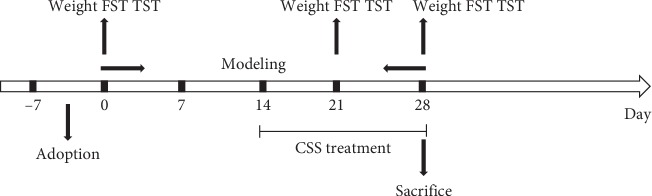
Timeline of the experiment. Mice were randomly divided into eight groups: In Liver-Qi stagnation: control, model, 4 g/kg of CSS and 12 g/kg of CSS; in liver stagnation and spleen deficiency: control, model, 4 g/kg CSS and 12 g/kg CSS. CSS or distilled water was given once per day 14 days after induction of depression. Weight, FST, and TST were assessed on days 0, 21, and 28. The mice were sacrificed, and liver microsomes were used to evaluate CYP450 expression after 28 days by Western blot analysis and RT-qPCR.

Mice were randomly assigned to 7 groups in a blinded manner (*n* = 10 per group): (1) control group, (2) LQS model group, (3) LSSD model group, (4) 4 g/kg CSS + LQS model group, (5) 12 g/kg CSS + LQS model group, (6) 4 g/kg + LSSD model group, and (7) 12 g/kg CSS + LSSD model group. Control group mice were housed five per cage. Mice from the other groups were individually caged. The herb-treated groups received CSS (4 g/kg, 12 g/kg) orally once per day from day 15 to day 28 of depression induction. Weight measurements and behaviour testing as well as data analyses were all completed by two investigators who were blinded to the experimental design. All animals were sacrificed after 2 weeks of treatment with CSS, and liver microsomes were prepared for analysis by WB and qRT-PCR (a diagram of timeline of the experiment is shown in Figure 1).

### 2.5. Models

With some modifications, the models were established as previously reported [16, 23]. The protocol for the LQS model was as follows: restraint stress was induced by placing mice individually in a ventilated 50 mL polypropylene tube (diameter of 3 cm and length of 10 cm, with a 1 cm hole in the cover for air diffusion) for 3 h every day for 28 days [24]. The protocol for the LSSD model was as follows: restraint stress was induced by using the above-mentioned device for 3 h, followed by exposing mice to mild swimming by placing them individually in a plastic cylinder (40 cm in diameter × 18 cm high) containing 10 cm deep water (25 ± 1°C) for 10 min and food deprivation for 24 h. These procedures were repeated for 28 days unless otherwise indicated.

### 2.6. Weight Measurement and Behaviour Testing

#### 2.6.1. Body Weight

Body weight was recorded on day 0 (before the beginning of the experiments), day 21, and day 28 of the experiments.

#### 2.6.2. Tail Suspension Test (TST)

As previously described [25], mice were separately suspended in a cage (25 cm × 25 cm × 30 cm) by their tails 15 cm above the cage bottom with adhesive tape on day 0, day 21, and day 28. The total duration of the immobile time measured over the last 4 min of the 6-min test period was the dependent variable. Mice crawling to the tail during the tests was eliminated from the tests.

#### 2.6.3. Forced Swimming Test (FST)

A protocol was adapted from our previous work [26]: mice were individually forced to swim for 6 min on day 0, day 21, and day 28 in a plastic cylinder (40 cm height × 18 cm diameter) filled with water (25 ± 1°C) up to 10 cm. The duration of immobility of each mouse was considered during the final 4 min by investigators who were blinded to the experimental design. Mice were defined as immobile when floating motionless or only making movements necessary to hold their head above the water surface. The decrease in the duration of immobility was used to assess antidepressant-like effects [27–29].

### 2.7. Preparation of Liver Microsomes

Microsomal fractions were prepared according to a published protocol [30–32]. Briefly, the treatments were terminated after two weeks (on day 28), and then, all animals were sacrificed by decapitation, and their livers were quickly removed and promptly washed with ice-cold distilled water, followed by ice-cooled 67 mM potassium phosphate buffer (pH 7.4) containing 1.15% (w/v) KCl. The livers were then was homogenized in 6 mL of ice-cold 10 mM PBS buffer (pH 7.4). The homogenate was centrifuged at 10,000 × g for 30 min at 4°C. The supernatant was further centrifuged at 100,000 × g for 60 min at 4°C. The pellet was resuspended in 10 mM fresh, ice-cold PBS buffer (pH 7.4) and was centrifuged at 100,000 × g for 60 min at 4°C. The final pellet was resuspended in 20% glycerol-PBS and was stored at −80°C before being used.

### 2.8. Plasmid Construction

The CYP3A4-XREM (xenobiotic responsive enhancer module)-Luc plasmid containing the proximal promoter (−362/+53) and distal XREM (−7836/−7208) inserted in the pGL4.17 vector (Promega) and the CYP3A4-pGL4.17-Luc luciferase reporter was constructed. The human PXR expression plasmid was donated by Professor Guo Wang (Institute of Clinical Pharmacology, Central South University, Changsha, China). All expression plasmids were sequence verified (the details are shown in Supplementary [Supplementary-material supplementary-material-1]).

### 2.9. Transfections and Luciferase Assays

HepG2 cells were grown in high sugar DMEM containing 10% fetal bovine serum. Caco2 and LS174T cells were grown in DMEM/F12 containing 10% fetal bovine serum. After the cells were seeded into 24-well plates at 2 × 105 cells per well, they were transfected with 600 ng/well of CYP3A4-Luc, 10 ng/well of pRL-SV40 (Promega) and 100 ng/well of pcDNA3.1-PXR or pcDNA3.1 mixed with 5 *μ*l Lipofectamine 2000 (Invitrogen) for 4 to 6 h [33]. Then, the cells were washed in PBS, which was subsequently replaced with DMEM containing 10% fetal bovine serum, before being treated with rifampicin, vehicle (DMSO, 0.1%), PCN or a range of concentrations of glycyrrhetinic acid (1 *μ*M, 10 *μ*M, 20 *μ*M) in triplicate for 24 h. Finally, luminescence and fluorescence were assayed as per the manufacturer's instructions.

### 2.10. Western Blot

The protein levels of CYP450 isoforms in the liver microsomes were estimated by Western blot analysis. Briefly, liver tissues were homogenized in RIPA lysis buffer containing a protease inhibitor. The homogenate was centrifuged for 15 min (12,000 r, 4°C). The supernatants were collected for analysis by a BCA assay. Microsomal proteins (10 mg per sample) were separated by SDS-PAGE and then transferred onto polyvinylidene difluoride membranes. After the membranes were incubated with 5% BSA for 2 h at room temperature, they were incubated with one of the following primary antibodies (Abcam, Cambridge, UK): mouse anti-CYP1A2 (ab22717, 1 : 1000); rabbit anti-CYP2C9 (ab4263, 1 : 1000); rabbit anti-CYP2C19 (ab137015, 1 : 5000); rabbit anti-CYP2D6 (ab62204, 1 : 500); rabbit anti-CYP2E1 (ab28146, 1 : 5000); rabbit anti-CYP3A4 (ab3572, 1 : 2000); or rabbit anti-GAPDH (Proteintech, 10494-1-AP, 1 : 5000) overnight at 4°C. Then, the blots were incubated with a secondary antibody (HRP goat antimouse IgG or HRP goat antirabbit IgG) for 2 h at room temperature. The immunopositive bands were visualized by a chemiluminescent substrate (Thermo Fisher, USA) and by the ChemiDoc XRS digital documentation system (Bio-Rad, Hercules, CA, USA). The amount of protein expression is presented relative to the levels of GAPDH.

### 2.11. qRT-PCR

Total RNA was obtained from tissues or LS174T cells in each group using Trizol (Invitrogen, Carlsbad, CA, USA). Then, reverse transcription was performed with a reverse transcription assay kit following the manufacturer's instructions (CoWin Biosciences, China). Amplification was performed using a SYBR PCR kit (Invitrogen, Carlsbad, CA, USA). The following thermocycling protocol was used: 95°C for 10 min, 40 cycles of 5 s at 95°C, 30 s at 60°C, and melting at 60°C. The mouse-specific primers for CYP1A2, CYP2C9, CYP2C19, CYP2D6, CYP3E1, CYP3A4, and GAPDH were designed with Premier 5.0 software (PRIMER Biosoft International, CA, USA) and made by Sangon (the sequences of the primers are shown in Table 2). Melting curves of all samples were generated as controls to test specificity. All gene expression data were calculated by 2^−ΔΔCT^.

### 2.12. Bioinformatics Analysis

To obtain the known bioactive chemical ingredients of 7 herbs of CSS, bioinformatics analysis was conducted as previously described [34, 35]. In brief, a total of 150 compounds (Supplementary [Supplementary-material supplementary-material-1]) with oral bioavailability (OB) ≥ 30% and a druglikeness index (DL) ≥ 0.18 as potential active compounds were derived from the TCMSP (Traditional Chinese Medicines for Systems Pharmacology) Database and Analysis Platform (http://lsp.nwu.edu.cn/tcmsp.php). We searched 279 candidate targets from the TCMSP and UniPort databases (Supplementary [Supplementary-material supplementary-material-1]) for all 150 compounds in CSS. The protocol for DAVID, KEGG, and Functional Annotation Bioinformatics Microarray Analysis (https://david.ncifcrf.gov/) was as follows: Step 1: enter the gene list with 279 targets. Step 2: select the identifier as the OFFICIAL-GENE-SYMBOL. Step 3: list the type as background. Step 4: select human as the background and then use the data from the Cytoscape software to build the network chart. Another method was used to confirm the results in BATMAN-TCM (http://bionet.ncpsb.org/batman-tcm/): Input “CHAI HU SHU GAN SAN” with a score cut-off of 20 and an adjusted *p* value cut-off of 0.05; then, the results of the target prediction and KEGG pathway analysis were available to build a network chart by the Cytoscape software.

### 2.13. Statistical Analysis

Statistical analyses were performed by using SPSS 22.0 (International Business Machines Corp, Armonk, NY, USA). All data are presented as the means ± SEM. Comparisons between multiple groups were performed by one-way analysis of variance (ANOVA) with the post hoc Tukey's test. Significance was accepted as *p* < 0.05.

## 3. Results

### 3.1. CSS Specifically Alleviates Depressive-Like Behaviour in LQS of Depression

**Figure 2:**
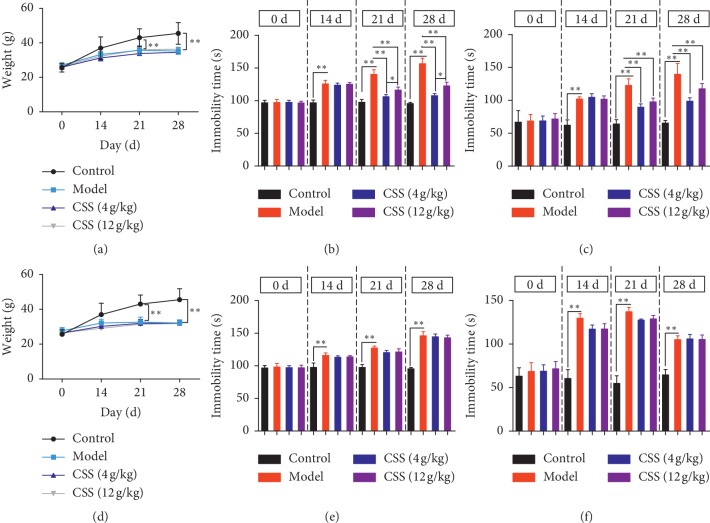
Effects of CCS on body weight and behaviour testing in mice exposed to stress, overfatigue, and an improper diet. (a, d) Effects of CSS (4 g/kg, 12 g/kg) on body weight in the LQS and LSSD depression models. (b, e) Effects of CSS (4 g/kg, 12 g/kg) on the immobility time in the two depression models in the forced swimming test. (c, f) Effects of CSS (4 g/kg, 12 g/kg) on the immobility time in the two depression models in the tail suspension test. ^*∗*^*p* < 0.05, ^*∗∗*^*p* < 0.001 compared with the depression model group.

As shown in Figures 2(a) and 2(d), there were no differences in body weight between groups before the experiment. After experiencing stress in two depressive models (the LQS and LSSD models) for three and four weeks, the body weights of the two model groups were measured to be lower than that of the control group. After 2 weeks of CSS treatment, no significant effects on body weight were observed in the two depressive model groups, even at a dose of up to 12 g/kg.

We conducted FST and TST to assess the antidepressant-like effects of CSS. Significant differences between the depressive model groups and the control group were observed. Compared with the control group, the immobility duration of the two depression model groups remarkably increased, which indicated that the models worked well (*p* < 0.001) (Figures 2(b)–2(f)). Furthermore, after the LQS model group was treated with CSS (4 g/kg and 12 g/kg), an evident decrease in immobility time was observed (*p* < 0.001) (Figures 2(b)–2(f)), while CSS had no influence on LSSD animals, which means that CSS had no antidepressant effects on LSSD of depression. These results suggested that CSS specifically attenuated the depressive-like behaviour of LQS depression.

### 3.2. CSS Markedly Regulates CYP450 Expression in Liver Microsomes of Depressive Mice with LQS

**Figure 3:**
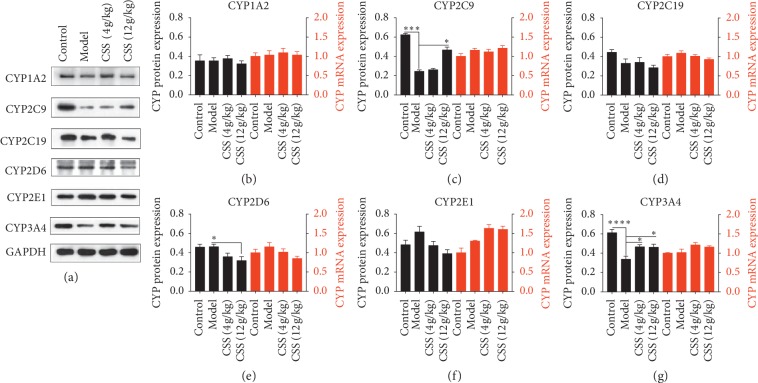
Effects of CSS on the proteins and mRNAs of CYP450 in the LQS depression model after CSS administration. (a) Effects of CSS on CYP450 proteins in the LQS depression model after 4 weeks of constraint stress and 2 weeks of CSS treatment. (b–f) Effects of CSS on the proteins (colour: blank) and mRNAs (colour: red) of CYP450 (CYP1A2, CYP2C9, CYP2C19, CYP2D6, CYP2E1, CYP3A4) in the LQS depression model after 2 weeks of CSS treatment. ^*∗*^*p* < 0.05, ^*∗∗*^*p* < 0.01, ^*∗∗∗*^*p* < 0.001, ^*∗∗∗∗*^*p* < 0.0001 compared with the depression model group.

**Figure 4:**
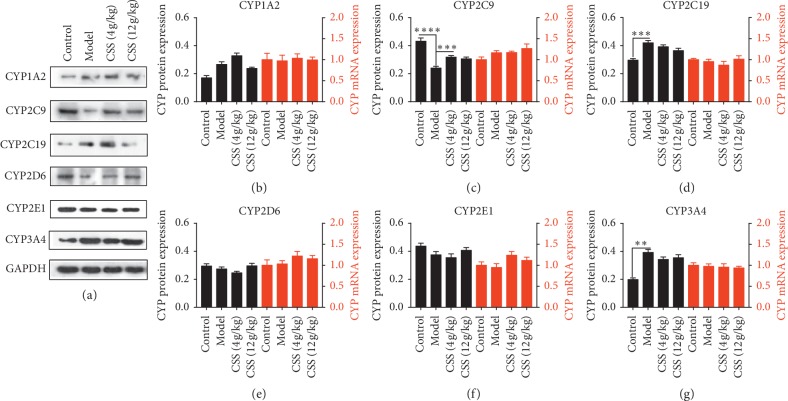
Effect of CSS on CYP450 proteins and mRNAs in the LSSD depression model after 4 weeks of constraint stress, overfatigue, and an improper diet. (a) Effects of CSS on CYP450 proteins in the LSSD depression model after 4 weeks of constraint stress, overfatigue, and an improper diet with 2 weeks of CSS treatment. (b–f) Effects of CSS on the proteins (colour: blank) and mRNAs (colour: red) of CPY450 (CYP1A2, CYP2C9, CYP2C19, CYP2D6, CYP2E1, CYP3A4) in the LSSD depression model with 2 weeks of CSS treatment. ^*∗*^*p* < 0.05, ^*∗∗*^*p* < 0.01, ^*∗∗∗*^*p* < 0.001, ^*∗∗∗∗*^*p* < 0.0001 compared with the depression model group.

To determine the association between the efficacy of CSS and CYP450 expression, we measured the expression levels of CYP450 enzymes. Lower protein expression levels of CYP2C9 and CYP3A4 (but not of CYP1A2, CYP2C19, CYP2D6, or CYP2E1) were observed in the LQS group compared with the control group (Figures 3(a)–3(g)). However, downregulated CYP2C9 protein expression and elevated CYP2C19 and CYP3A4 protein expression were detected in the LSSD group compared with the control group (Figures 4(a)–4(g)). Intriguingly, CSS significantly increased the level of CYP2C9 in both the LQS and LSSD groups (Figures 3(c) and 4(c)), while elevated CYP3A4 protein was only observed in the LQS group (Figure 3(g)). These findings demonstrated that CYP3A4 could be a specific CYP450 enzyme responsible for the antidepressive effects of CSS in LQS.

The effects of CSS on the mRNA of CYP450 enzymes were also examined in liver microsomes. Unexpectedly, the expression level of CYP450 enzyme mRNA in the depressive models was not significantly different from that of the control group. Additionally, after exposure to CSS (4 g/kg and 12 g/kg), CYP mRNA expression did not change (Figures 3 and 4).

The results indicate that posttranscriptional or posttranslational modifications occur to CYP450 produced in the liver, consistent with previous research [36].

### 3.3. Bioinformatics Results Imply That Glycyrrhetinic Acid Is Involved in the Specific Regulation by CSS

**Figure 5:**
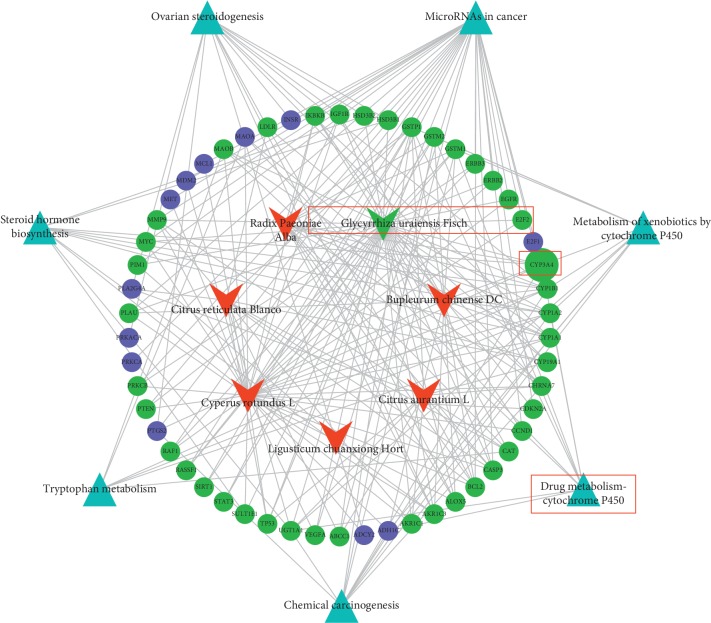
Schematic of the bioinformatics analysis of TCMSP combined with UniProt, DAVID, and KEGG analyses to explore the potential representative compounds of CSS.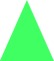: Pathways related to CYP450. 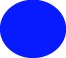: Targets related to CSS. 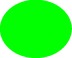: Targets related to GA in CSS. 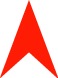: Herbs in CSS.

The sophisticated compounds of CSS make it difficult to elucidate the therapeutic mechanisms of CYP3A4 regulation following depression. In this study, we explored the TCMSP, UniProt, and DAVID databases. We found that 54/279 targets in CSS interacted with CYP450-related pathways: Metabolism of xenobiotics by cytochrome P450, drug metabolism—cytochrome P450, chemical carcinogenesis, tryptophan metabolism, steroid hormone biosynthesis, ovarian steroidogenesis, and microRNAs. Among the 54 targets, Glycyrrhiza uralensis Fisch (42/54) had the largest number of interactions, followed by Cyperus rotundus L (37/54), Bupleurum chinense DC (18/54), Radix Paeoniae Alba (15/54), Citrus aurantium L (13/54), Citrus reticulata Blanco (9/54), and Ligusticum chuanxiong Hort (1/54) (Figure 5, Supplementary [Supplementary-material supplementary-material-1]-[Supplementary-material supplementary-material-1]). The data were also confirmed in BATMAN-TCM (http://bionet.ncpsb.org/batman-tcm) (Supplementary [Supplementary-material supplementary-material-1]). Moreover, previous studies showed that glycyrrhetinic acid (GA) was the bioactive compound after oral administration of Glycyrrhiza uralensis Fisch [37].

### 3.4. GA Promotes CYP3A4 Expression by the Nuclear Receptor PXR *in vitro*

**Figure 6:**
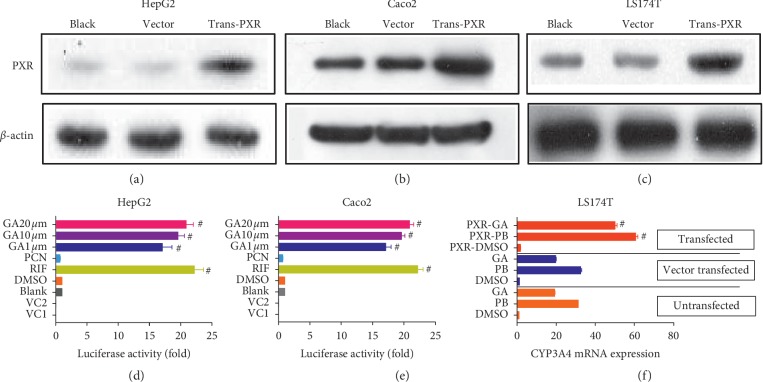
Effects of GA on CYP3A4 expression via PXR *in vitro*. Blank: cells not transfected. Vector: cells transfected with 600 ng CYP3A4, 100 ng pcDNA3.1, and 10 ng PRL-SV40. Trans-PXR: cells transfected with 600 ng CYP3A4, 100 ng pcDNA3.1-PXR, and 10 ng PRL-SV40. Western blot verification of transfection of the PXR plasmid into HepG2 (a), Caco2 (b), and LS174T (c) cells. (d, e) VC1: cells were transfected with pcDNA3.1-PXR, PRL-SV40, and pGL4.17. VC2: cells were transfected with pcDNA3.1, PRL-SV40, and pGL4.17-CYP3A4. DMSO. The RIF, PCN, and GA (1, 10, 20 *μ*M) groups were transfected with pcDNA3.1-PXR, PRL-SV40, and pGL4.17-CYP3A4. (f) CYP3A4 mRNA expression in PXR-mediated LS174T cells. ^#^*p* < 0.001 compared with DMSO or PXR-DMSO.

We addressed whether GA influenced PXR-mediated transcriptional activation of CYP3A4. We successfully transfected the PXR plasmid into HepG2 and Caco2 cells (Figures 6(a) and 6(b)). Rifampicin (10 *μ*M, hPXR agonist) or vehicle (DMSO, 0.1%), PCN (10 *μ*M, rPXR agonist), and a range of concentrations of glycyrrhetinic acid (1 *μ*M, 10 *μ*M, 20 *μ*M) were provided for 24 h; then, luciferase activity was assessed. As shown in Figures 6(d) and 6(e), GA remarkably increased PXR-mediated activation of CYP3A4 luciferase activity in HepG2 and Caco2 cells compared with the DMSO group in a dose-dependent manner (*p* < 0.001), and GA (20 *μ*M) increased PXR-mediated activation of CYP3A4 luciferase activity in HepG2 and Caco2 cells, which was equal to the effect of rifampicin.

In addition, PXR-mediated CYP3A4 mRNA expression was studied in LS174T cells. We successfully transfected the PXR plasmid into LS174T cells (Figure 6(c)). As shown in Figure 6(f), GA increased PXR-mediated CYP3A4 mRNA expression compared with the PXR-mediated DMSO group (*p* < 0.001), but the effect was inferior to that of phenobarbital (PB, the activator of PXR). These data indicated that GA increased PXR-mediated CYP3A4 expression.

## 4. Discussion

**Figure 7:**
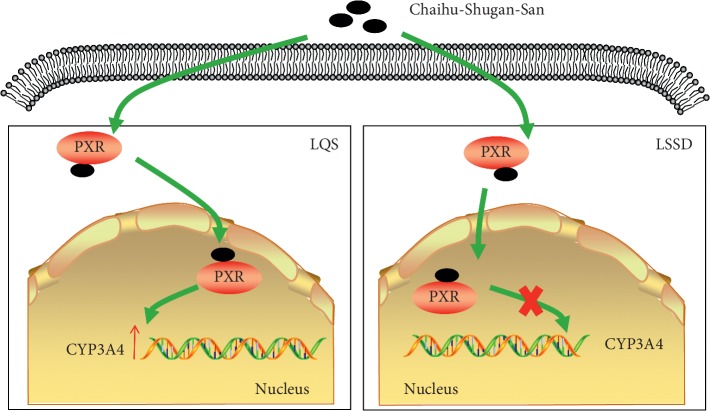
CSS activates PXR and binds to the CYP3A4 transcriptional region to increase CYP3A4 expression in LQS-associated rather than in LSSD-associated depression.

To the best of our knowledge, this is the first report on the specific reinforcement of CYP3A4 translation via the nuclear receptor PXR induced by CSS to treat LQS of depression. Our work shows that CSS uniquely improves the depressive-like behaviour of LQS and recovers CYP3A4 protein expression in LQS animals. The bioinformatics analysis results reveal that the unique regulation of CYP3A4 mainly refers to the action of GA, which is a bioactive compound from CSS. The following *in vitro* work confirms that GA increases CYP3A4 expression through the nuclear receptor PXR (Figure 7).

As a classic antidepressant formula, CSS has been used in previous studies [10–14, 38]. However, traditional Chinese syndrome differentiation is the core of TCM theory. Therefore, in this study, using LQS and LSSD as depressive models, we sought to explore the specific effects of CSS on depression and its potential mechanisms in mouse models. We found that CSS ameliorated the depression-like symptoms and upregulated the CYP3A4 protein in LQS but not in LSSD, suggesting that CYP3A4 could be responsible for the syndrome-specific effects of CSS on LQS-mediated depression. However, as a classic herbal medicine, CSS is a complicated multicomponent system that tends to interact with multiple targets and regulate multiple pathways throughout the human body. It is important to determine the ingredients that contribute to the activation of CYP3A4 in CSS.

In recent years, TCMSP and bioinformatics analysis tools, including the UniProt, DAVID and BATMAN-TCM databases, have been used to perform TCM ingredient target prediction and subsequent network pharmacology [39, 40]. For example, the therapeutic mechanism (multicomponent, multitarget, and multipathway) of Qi-shen-Yi-qi dripping pill (QSYQ) was explored by BATMAN-TCM [41]. In addition, systems polypharmacology approaches (including TCMSP databases) were applied to probe the definite mechanisms of liquorice [34]. Furthermore, a systems pharmacology approach was used to investigate the molecular mechanisms of the representative Lonicera japonica and Fructus Forsythiae in influenza disease prevention and treatment [35]. In this work, we performed bioinformatics analysis (including the TCMSP, UniProt, DAVID, KEGG, and BATMAN-TCM databases). After using TCMSP and UniProt to obtain targets of compounds from CSS, we analysed the targets with respect to CYP450 using the DAVID database. We found that Glycyrrhiza uralensis Fisch had the largest number of interactions with pathways related to CYP450. Given that GA is absorbed in blood as the active metabolite of Glycyrrhiza uralensis Fisch, GA was used for confirmation *in vitro.*

CYP450 enzymes serve as the major enzyme system that regulates TCM metabolism [36, 42, 43]. Among this superfamily, CYP3A4 is a major isozyme that is highly expressed in the liver and is known to metabolize many different drugs; CYP3A4 is regulated by pregnane X receptor (PXR) [44, 45]. A number of therapeutic agents, including TCM, are the main substrates of CYP3A4. Curcumin [46], Oldenlandia diffusa [19], and glycyrrhizin [9] are prominent examples from herbal drugs that have been reported to have CYP3A4 induction effects. Our data suggested that CSS increased CYP3A4 protein expression *in vivo*, and GA was predicted and demonstrated to activate CYP3A4 transcription *in vitro*. These results were in agreement with previous studies. Nevertheless, the role of GA on CYP3A4 induction in our research was found to be different from that reported in a previous study [47]. Our data appeared to show that CSS increased CYP3A4 protein expression. GA, as a bioactive compound of CSS, was shown to activate CYP3A4 transcription. Compared with a previous report, we used the LQS and LSSD depression models in mice, but the other study used normal rats. We used transfection and the luciferase assay with cells, while liver microsomes were used in the previous study *in vitro*. Additionally, even though a substantial portion of CSS that affects the activity of CYP3A4 has been genetically determined, it can also be affected by intrinsic factors, such as age, gender, and comorbidity [48]. All of these factors could be responsible for the contradiction.

This study has several limitations. Although we confirmed that CSS increases CYP3A4 expression via the pregnane X receptor in the LQS of depression mouse model, the mechanisms of CSS in depressive patients remain unclear due to species differences between rodents and humans. Furthermore, CYP3A4 regulation by GA was via PXR in our study, but other nuclear receptors, such as androstane receptor (CAR), should be explored. Additional studies are required to elucidate the mechanisms underlying these phenomena in the future.

## 5. Conclusions

This work shows that CSS specifically increases CYP3A4 translation via the nuclear receptor PXR in LQS of depression. This activation is related to the bioactive GA. This work provides novel insight into Chinese syndrome-based therapy for depression.

## Figures and Tables

**Table 1:** Herbs and their component ratios in Chaihu-Shugan-San.

English name	Chinese name	Ratio
Bupleurum chinense DC	Chai hu	4
Radix Paeoniae Alba	Bai shao	3
Ligusticum chuanxiong Hort	Chuan xiong	3
Citrus aurantium L	Zhi ke	3
Cyperus rotundus L	Xiang fu	3
Citrus reticulata Blanco	Chen pi	4
Glycyrrhiza uralensis Fisch	Gan cao	1

**Table 2:** Summary of the qRT-PCR primer sequences.

Gene	Primers	Sequences	Product length
CYP1A2	Forward	ACAAGACCCAGAGCGAGAAG	108 bp
	Reverse	GCAGCAGGATGGCTAAGAAG	
CYP2C9	Forward	TTCCTCTTCCAGCAAACTCC	120 bp
	Reverse	TTTCTGCCAATCACACGTTC	
CYP2C19	Forward	ACATCTGCCAATCCTTCACC	150 bp
	Reverse	TTCCTCTTCCAGCAAACTCC	
CYP2D6	Forward	GGTAGGGTCCCAGGTCGTCT	226 bp
	Reverse	CTATGCCTGCCGCTTTGAGT	
CYP2E1	Forward	TTTCCCTAAGTATCCTCCGTGAC	193 bp
	Reverse	TCGTAATCGAAGCGTTTGTTG	
CYP3A4	Forward	GCCACTCACCCTGATGTCC	117 bp
	Reverse	CACCACCATGTCAAGATACTCC	
GAPDH	Forward	CGGCAAATTCAACGGCACA	86 bp
	Reverse	GGTCTCGCTCCTGGAAGATGG	
*β*-actin	Forward	CATTGTGATGGACTCCGGAGACGG	116 bp
	Reverse	CATCTCCTGCTCGAAGTCTAGAGC	

## Data Availability

The data used to support the findings of this study are included within the article.

## Supplementary Materials

Supplementary Materials Supplementary file 1: the quality control of CSS by using UPLC. Supplementary file 2: sequencing verification; the expression plasmids (PXR and CYP3A4) were sequence verified by DNA sequencing. Supplementary file 3: the compounds of herbs of CSS with oral bioavailability (OB) ≥ 30% and druglikeness index (DL) ≥ 0.18 as potential active compounds were derived from the database TCMSP. Supplementary file 4: the candidate targets for all the compounds in CSS from TCMSP and UniPort databases and KEGG pathway enrichment analysis result of CSS. Figure S5: bioinformatics analysis by BATMAN-TCM combined with KEGG to obtain the potential representative compounds of CSS in LQS of depression. Table S5: KEGG pathway enrichment analysis result of CSS by BATMAN-TCM.
