# Supplementary material for: Chaihu-Shugan-San Reinforces CYP3A4 Expression via Pregnane X Receptor in Depressive Treatment of Liver-Qi Stagnation Syndrome
Source: Evid Based Complement Alternat Med. 2019 Oct 31;2019:9781675. doi: 10.1155/2019/9781675 (PMC6875207; doi:10.1155/2019/9781675)
Supplement: Supplementary Materials — Supplementary file 1: the quality control of CSS by using UPLC. Supplementary file 2: sequencing verification; the expression plasmids (PXR and CYP3A4) were sequence verified by DNA sequencing. Supplementary file 3: the compounds of herbs of CSS with oral bioavailability (OB) ≥ 30% and druglikeness index (DL) ≥ 0.18 as potential active compounds were derived from the database TCMSP. Supplementary file 4: the candidate targets for all the compounds in CSS from TCMSP and UniPort databases and KEGG pathway enrichment analysis result of CSS. Figure S5: bioinformatics analysis by BATMAN-TCM combined with KEGG to obtain the potential representative compounds of CSS in LQS of depression. Table S5: KEGG pathway enrichment analysis result of CSS by BATMAN-TCM. [file 9781675.f1.zip › 9781675.f1/supplementary file 1.docx]

**Supplementary file 1 :**

A）

B）

40.616

31.625

9

26.399

20.751

7

20.418

7.235

4

13.501

（a）

12

11

37.617

10

5

6

8

18.914

19.964

3

2

6.465

1

5.393

12

40．614

26．415

20.727

8

7

20.393

6

19.938

18.879

5

3

7.185

（b）

11

9

31．610

4

13．448

2

1

6.430

5.361

The quality control of CSS by using UPLC. As previously described [1], Chromatographic separation was performed on an Acquity UPLC BEH 2.1 × 50 mm id, 1.7 μm C18 column (Waters Corporation using an Acquity UPLC system equipped with an Acquity photo‐diode array detector ). The wavelength was 252 nm in the UV spectrum. The volume of sample injected was 3 μL. The flow rate and operating temperature were maintained at 0.5 mL/min and 40 ℃, respectively.

(A) Standard chromatogram of a blank plasma sample. (B) Chromatogram of a plasma sample of mice with depression taken 30 min after oral administration of CSS. Chromatogram of (1) Peony lactone glycosides, (2) Ferulic acid, (5) Naringin, (6) Hesperidin, (11) A-flavonoids, (12) Licorice subacid.

Reference

[1] Yang Wang, Rong Fan, Jiekun Luo, et.al. An ultra high performance liquid chromatography with tandem mass spectrometry method for plasma and cerebrospinal fluid pharmacokinetics of rhein in patients with traumatic brain injury after administration of rhubarb decoction. *Journal of Separation Science* , 2015;38(7):1100-8.
