# Supplementary material for: Chaihu-Shugan-San Reinforces CYP3A4 Expression via Pregnane X Receptor in Depressive Treatment of Liver-Qi Stagnation Syndrome
Source: Evid Based Complement Alternat Med. 2019 Oct 31;2019:9781675. doi: 10.1155/2019/9781675 (PMC6875207; doi:10.1155/2019/9781675)

**Sequencing verification**

The expression plasmids (PXR and CYP3A4 ) were sequence verified by DNA sequencing

1. Results from PXR shown in the form of a chromatogram. The colors represent the four bases: blue is C, green is A, black is G and red is T.


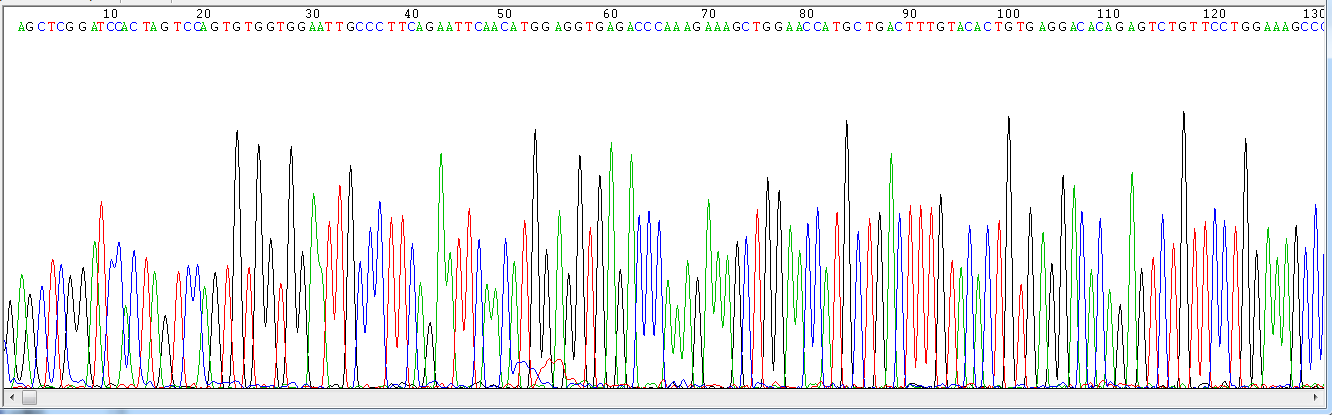


1. Sequencing comparison of the donated PXR plamid and design before sequencing


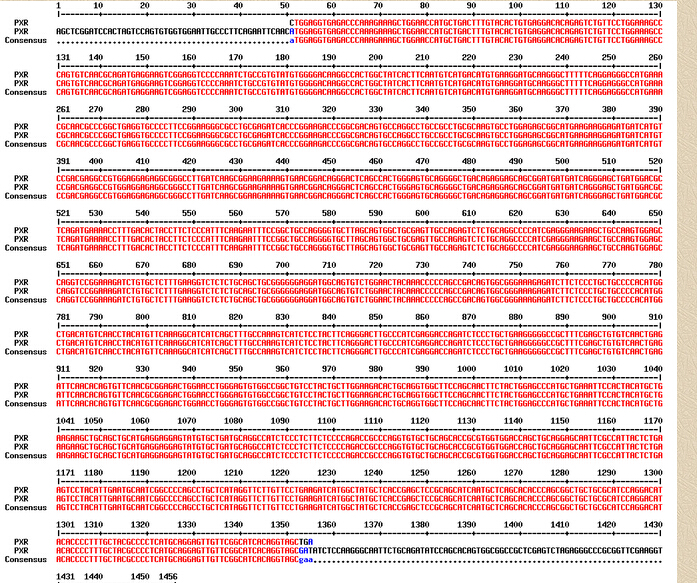


The result of PXR plasmid Sequence is as follows:


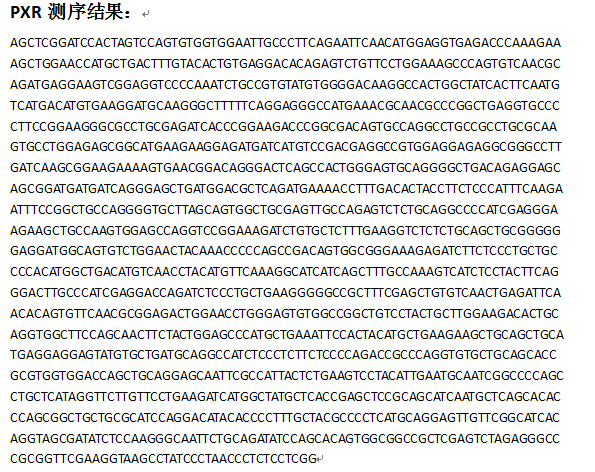


CYP3A4

1. Results from CYP3A4 shown in the form of a chromatogram. The colors represent the four bases: blue is C, green is A, black is G and red is T.


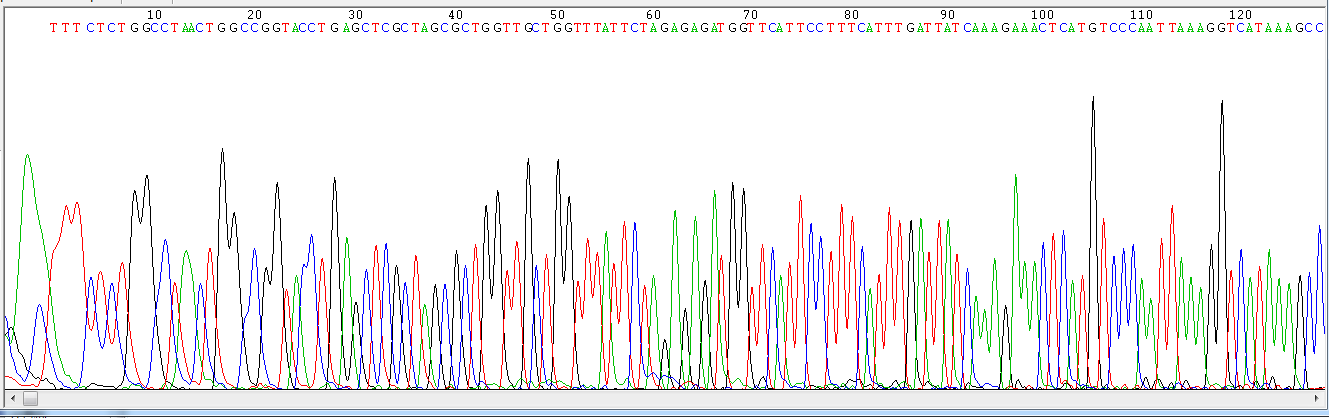


1. Sequencing comparison of the CYP3A4 plamid we constructed and design before sequencing


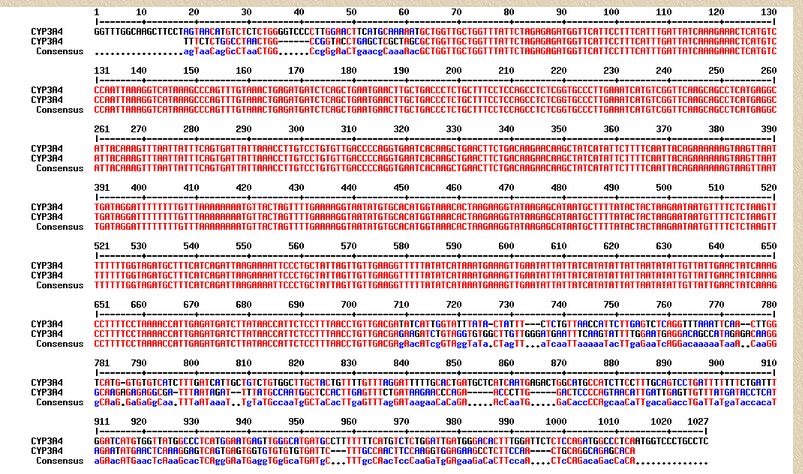


The result of CYP3A4 plasmid Sequence is as follows :


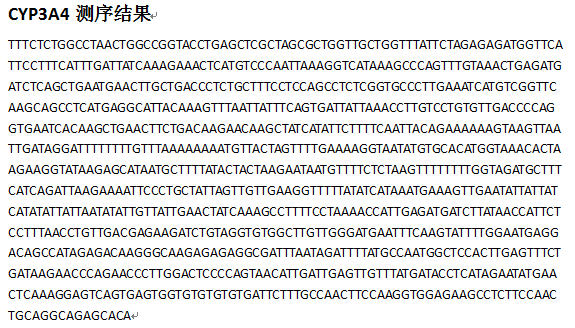

Supplement: Supplementary Materials — Supplementary file 1: the quality control of CSS by using UPLC. Supplementary file 2: sequencing verification; the expression plasmids (PXR and CYP3A4) were sequence verified by DNA sequencing. Supplementary file 3: the compounds of herbs of CSS with oral bioavailability (OB) ≥ 30% and druglikeness index (DL) ≥ 0.18 as potential active compounds were derived from the database TCMSP. Supplementary file 4: the candidate targets for all the compounds in CSS from TCMSP and UniPort databases and KEGG pathway enrichment analysis result of CSS. Figure S5: bioinformatics analysis by BATMAN-TCM combined with KEGG to obtain the potential representative compounds of CSS in LQS of depression. Table S5: KEGG pathway enrichment analysis result of CSS by BATMAN-TCM. [file 9781675.f1.zip › 9781675.f1/supplementary file 2.docx]
