# Supplementary material for: Chaihu-Shugan-San Reinforces CYP3A4 Expression via Pregnane X Receptor in Depressive Treatment of Liver-Qi Stagnation Syndrome
Source: Evid Based Complement Alternat Med. 2019 Oct 31;2019:9781675. doi: 10.1155/2019/9781675 (PMC6875207; doi:10.1155/2019/9781675)
Supplement: Supplementary Materials — Supplementary file 1: the quality control of CSS by using UPLC. Supplementary file 2: sequencing verification; the expression plasmids (PXR and CYP3A4) were sequence verified by DNA sequencing. Supplementary file 3: the compounds of herbs of CSS with oral bioavailability (OB) ≥ 30% and druglikeness index (DL) ≥ 0.18 as potential active compounds were derived from the database TCMSP. Supplementary file 4: the candidate targets for all the compounds in CSS from TCMSP and UniPort databases and KEGG pathway enrichment analysis result of CSS. Figure S5: bioinformatics analysis by BATMAN-TCM combined with KEGG to obtain the potential representative compounds of CSS in LQS of depression. Table S5: KEGG pathway enrichment analysis result of CSS by BATMAN-TCM. [file 9781675.f1.zip › 9781675.f1/supplementary file 5.docx]

**Fig.S5**


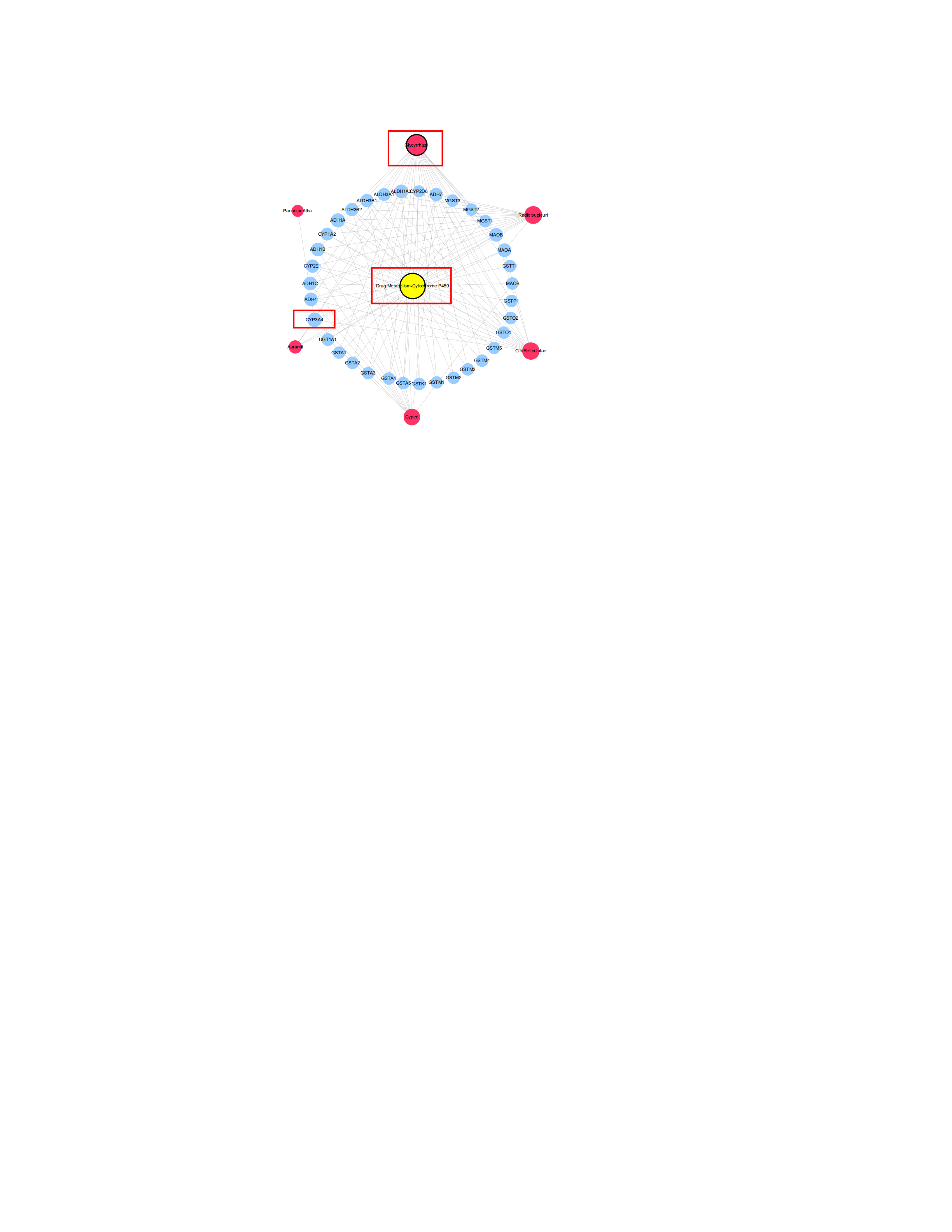


**Fig. S5.** Bioinformatics analysis by BATMAN-TCM combined with KEGG to obtain the potential representative compounds of CSS in LQS of depression. Yellow : pathways related to CYP450. Blue : targets related to CSS. Rose: herbs in CSS.

**Table. S5**  KEGG pathway enrichment analysis result of CSS by BATMAN-TCM.

| Compounds | KEGG pathway | Targets | P-value |
| --- | --- | --- | --- |
| CSS | Drug Metabolism-Cytochrome P450 | ADH1A;ADH1B;ADH1C;ADH4;ADH7;ALDH1A3;ALDH3A1;ALDH3B1;ALDH3B2;CYP1A2;CYP2D6;CYP2E1;CYP3A4;GSTA1;GSTA2;GSTA3;GSTA4;GSTA5;GSTK1;GSTM1;GSTM2;GSTM3;GSTM4;GSTM5;GSTO1;GSTO2;GSTP1;GSTT1;MAOA;MAOB;MGST1;MGST2;MGST3;UGT1A1(34 targets) | ＜0.00001 |
| Chaihu | Drug Metabolism-Cytochrome P450 | ADH1A;ADH1B;ADH1C;ADH4;ADH7;ALDH1A3;ALDH3A1;ALDH3B1;ALDH3B2;CYP1A2;CYP2E1;CYP3A4;MAOA;MAOB;UGT1A1(15targets) | 0.049 |
| Baishao | Drug Metabolism-Cytochrome P450 | CYP3A4(1 target) | 0.726 |
| Chenpi | Drug Metabolism-Cytochrome P450 | ADH1A;ADH1B;ADH1C;ADH4;ADH7;ALDH1A3;ALDH3A1;ALDH3B1;ALDH3B2;CYP1A2;CYP2E1;CYP3A4;MAOA;MAOB;UGT1A1(15targets) | 0.00023 |
| Zhike | Drug Metabolism-Cytochrome P450 | ALDH1A3;CYP3A4;MAOA;MAOB(4 targets) | 0.708 |
| Chuanxiong | - | - | - |
| Gancao | Drug Metabolism-Cytochrome P450 | ADH1A;ADH1B;ADH1C;ADH4;CYP3A4;GSTA1;GSTA2;GSTA3;GSTA4;GSTA5;GSTK1;GSTM1;GSTM2;GSTM3;GSTM4;GSTM5;GSTO1;GSTO2;GSTP1;GSTT1;MAOA;MAOB;MGST1;MGST2;MGST3(25targets) | ＜0.00001 |
| Xiangfu | Drug Metabolism-Cytochrome P450 | ADH1A;ADH1B;ADH1C;ADH4;ADH7;ALDH1A3;ALDH3B1;ALDH3B2;CYP2E1;CYP3A4; MAOB(11targets) | 0.0034 |
|  |  |  |  |
